# Monitoring the Burden of *Staphylococcus aureus*: A Multi-Year Retrospective Study Using Routine Laboratory Data from a Slovak Hospital

**DOI:** 10.3390/antibiotics15050443

**Published:** 2026-04-29

**Authors:** Andrej Minich, Peter Sabaka, Vladimír Heger, Rudolf Kubička, Peter Mihalov, Ján Jurenka, Ľubomír Soják, Juliana Pašková, Ľubica Slimáková, Romana Kalianková Chovanová, Michal Kajsik

**Affiliations:** 1 MediXbank, n.o., Novozámocká 1/67, 949 05 Nitra, Slovakia; romana.chovanova@medirex.sk (R.K.C.); michal.kajsik@gmail.com (M.K.); 2 Faculty of Medicine, Comenius University in Bratislava, Špitálska 24, 813 72 Bratislava, Slovakia; peter.sabaka@gmail.com (P.S.); mihalovpeter@gmail.com (P.M.); jan.jurenka@gmail.com (J.J.); 3 Heger s.r.o., Doľany 398, 900 88 Doľany, Slovakia; vladimir.heger@zdravotnykompas.sk; 4 Neoxx JSC, Ružová dolina 8, 821 09 Bratislava, Slovakia; rudolf.kubicka@neoxx.sk; 5 University Hospital Bratislava, Limbová 5, 831 01 Bratislava, Slovakia; lubosojak@zoznam.sk (Ľ.S.); julianapaskova001@gmail.com (J.P.); lubica.slimakova.ls@gmail.com (Ľ.S.)

**Keywords:** *Staphylococcus aureus*, multidrug resistance, data analysis

## Abstract

**Background/Objectives**: *Staphylococcus aureus* is one of the leading causes of bacterial infection-related mortality worldwide, with outcomes complicated by antimicrobial resistance; asymptomatic colonization (~30% of the population) increases the risk of subsequent infection, often with the colonizing strain. Comprehensive data from LMICs are lacking, and the COVID-19 pandemic has significantly influenced the incidence and epidemiology of *S. aureus* infections, highlighting a critical data gap in Slovakia. **Methods**: We conducted data analysis using the KNIME Analytics Platform (Zurich, Switzerland), an open-source, visual workflow environment that facilitates integration, preprocessing, and advanced analysis. This study analyzed 5 years of data from routine laboratory diagnostics extracted from the laboratory information system (LIS). **Results**: Our data reveals that the incidence of multidrug-resistant *S. aureus*, including MRSA, increased during 2020–2022—particularly in surgical departments, where MDR MRSA infection reached up to 12.5 per 1000 discharges—and remained elevated into the post-pandemic period (2023–2025). In contrast, MSSA incidence was consistently higher overall, with colonization rates reaching up to 71 per 1000 discharges in non-surgical departments, and was predominantly driven by colonization rather than infection. **Conclusions**: This study provides essential insights into the use of big data analytics platforms. Identified gaps, such as information about the difference between colonization vs. infection, and their implementation in the future, together with whole-genome sequencing, set a foundation for epidemiological research purposes in Slovakia.

## 1. Introduction

*Staphylococcus aureus*, including both methicillin-sensitive (MSSA) and methicillin-resistant (MRSA), remains a significant pathogen in European healthcare settings [1]. These bacteria can cause a range of infections, from minor skin and soft tissue infections to severe diseases like bacteremia, endocarditis, and osteomyelitis. The transmission of MRSA within healthcare facilities, especially in intensive care units, remains a persistent challenge [2,3]. While the incidence of hospital-acquired MRSA infections has witnessed a decline in certain European regions, thanks to effective infection control measures, community-acquired MRSA continues to pose a substantial public health threat [4,5,6].

The occurrence of hospital-acquired *S. aureus* infection adversely affects patient prognosis [7,8]. The outcomes are even worse if the pathogen is methicillin-resistant [9]. The nosocomial MRSA infections also pose a significant economic burden [10,11]. Multi-resistance, where bacteria develop resistance to multiple antibiotic classes, has been surging. The overuse of antibiotics, both in medical practice and agriculture, is a cardinal driver of this phenomenon. The European Centre for Disease Prevention and Control (ECDC) has been monitoring antimicrobial resistance trends and consistently warning about the consequences of antibiotic overuse [12]. European initiatives against the threat of antibiotic resistance have not been confined to surveillance. Research and development efforts are ongoing to discover novel antibiotics and alternative therapeutic strategies [13,14].

The resistance patterns vary not only among and within different bacterial species but also across geographical regions. Consequently, robust surveillance of various bacterial pathogens is essential for developing effective strategies and interventions to combat the emergence and spread of antibiotic resistance [15]. Slovakia (Central Europe) faces significant challenges related to antimicrobial resistance. Among 204 countries, Slovakia ranks 56th in age-standardized mortality rate per 100,000 population associated with AMR [16]. Slovakia has consistently exceeded the EU average. At the country level, eight countries in the EU had increases in hospital antibiotic consumption between 2019 and 2020, and 11 had increases between 2020 and 2021. Five countries (Bulgaria, Croatia, Greece, Portugal, and Slovakia) had higher hospital consumption in 2021 than in 2019 [12].

The COVID-19 pandemic substantially influenced the epidemiology of *Staphylococcus aureus*, including both MRSA and MSSA, although reported effects have been heterogeneous across settings. While some regions observed a temporary decline in community-associated *S. aureus* transmission following the implementation of non-pharmaceutical interventions (e.g., enhanced hygiene and reduced social contact) [17], multiple surveillance studies reported increased rates of hospital-onset MRSA bacteremia during the pandemic, particularly among patients with COVID-19 and in intensive care settings [18,19]. In parallel, increased empirical use of broad-spectrum antibiotics and disruptions in antimicrobial stewardship programs may have contributed to selective pressure favoring antimicrobial resistance [20,21]. Overall, the pandemic appears to have differentially affected MRSA/MSSA prevalence and multidrug resistance patterns in community versus hospital environments, with long-term consequences still requiring further evaluation.

An increasing number of diverse data sources are now being integrated for epidemiological analysis, allowing researchers to combine evidence from multiple origins [22]. Incorporating real-time information and big data analytics into public health systems strengthens preventive strategies and enables more effective outbreak control measures. By leveraging these tools, public health organizations can improve forecasting and management of such events, thereby mitigating their impact on human lives and the economy [23].

Systematically collecting and analyzing data and regular data monitoring allow for the identification of emerging resistance patterns, high-risk areas, specific multidrug-resistant (MDR) strains and the effect of the epidemiological approach. However, the efforts to combat AMR are challenged by the poor availability of reliable data, issues of integration, privacy, scalability, and data quality [24,25], particularly from low- and middle-income countries.

## 2. Results

This section presents the results of the longitudinal analysis of *S. aureus* derived from routine laboratory diagnostics and hospital activity data. First, the analytical framework developed for standardized data processing and surveillance is described to provide context for the subsequent findings. This is followed by an overview of the study dataset and temporal changes in hospital activity. Finally, incidence trends of *S. aureus* are reported after normalization to hospitalizations, stratified by resistance phenotype, specimen classification, and department category.

### 2.1. KNIME-Based Workflow for Longitudinal Surveillance

**Figure 1:**
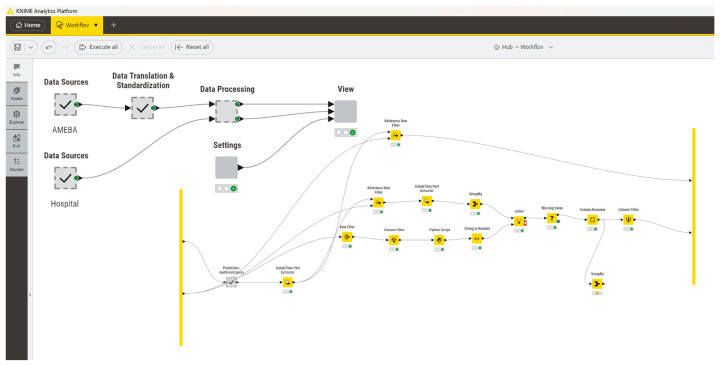
KNIME-based analytical workflow illustrating automated classification of *Staphylococcus aureus* isolates, department stratification, and normalization of incidence to completed hospitalizations. Arrows indicate the direction of data flow between nodes. The blue square represents a processing node, while green and red circles denote node status (green = successfully executed, red = error or not executed). Red symbols highlight nodes requiring attention or indicating potential issues.

To enable standardized and reproducible analysis of *S. aureus* burden from routine laboratory diagnostics, a comprehensive analytical workflow was developed within the KNIME Analytics Platform (Figure 1). The workflow integrates microbiological data with hospital activity data and applies a unified preprocessing strategy across the entire study period.

### 2.2. Characteristics of the Study Dataset and Hospital Activity

After data cleaning, expert-rule-based classification, and deduplication were implemented within the KNIME analytical workflow, the final dataset comprised 127,724 antibiotic susceptibility testing (AST) measurements corresponding to 9186 unique *Staphylococcus aureus* isolates collected between 2019 and 2025 (Table 1).

The annual number of unique *Staphylococcus aureus* isolates ranged from 1120 in 2024 to 1384 in 2022. The annual total number of AST measurements ranged from 13,062 in 2025 to 19,487 in 2022. Total completed hospitalizations decreased from 18,514 in 2019 to 11,295 in 2025, with consistently higher numbers in non-surgical than in surgical departments throughout the study period.

**Figure 2:**
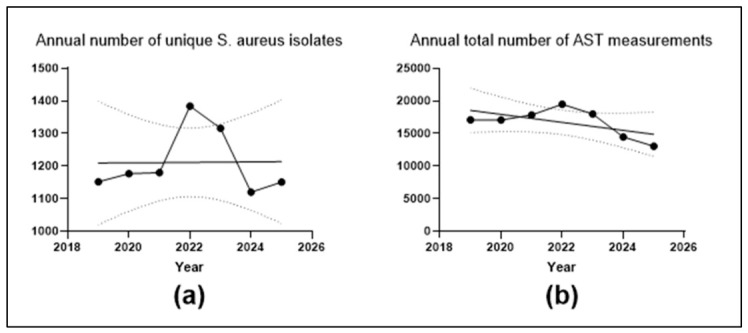
Annual numbers of unique *Staphylococcus aureus* isolates and antimicrobial susceptibility testing (AST) measurements during 2019–2025. (**a**) Annual number of unique *S. aureus* isolates. (**b**) Annual total number of AST measurements. Solid lines indicate simple linear regression fits; dotted lines indicate 95% confidence bands.

As shown in Figure 2, the annual number of unique *S. aureus* isolates varied within a relatively narrow range across the study period despite a peak in 2022. No significant linear trend was observed for the annual number of unique isolates (slope 0.68 isolates/year; 95% CI −51.83 to 53.19; *p* = 0.9748). Likewise, the annual total number of AST measurements increased up to 2022 and declined thereafter, but the overall linear trend was not statistically significant (slope −612.1 measurements/year; 95% CI −1553.0 to 328.7; *p* = 0.1553).

**Figure 3:**
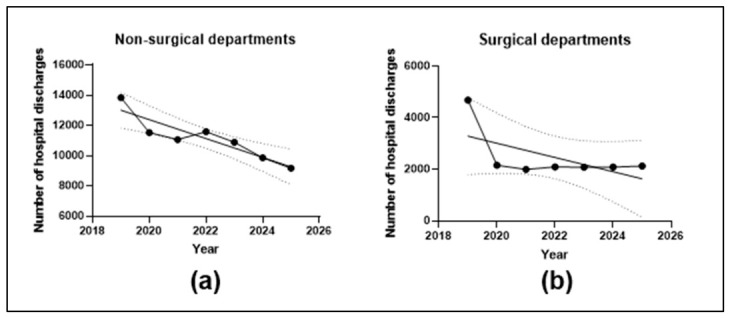
Annual numbers of completed hospitalizations by department type during 2019–2025. (**a**) Non-surgical departments. (**b**) Surgical departments. Solid lines indicate simple linear regression fits; dotted lines indicate 95% confidence bands.

Figure 3 summarizes annual hospital activity according to department type. In non-surgical departments, completed hospitalizations declined from 13,839 in 2019 to 9174 in 2025, corresponding to a significant downward linear trend (slope −625.0 discharges/year; 95% CI −951.8 to −298.2; *p* = 0.0044). In surgical departments, completed hospitalizations decreased from 4675 in 2019 to 2121 in 2025; however, the overall linear trend was not statistically significant (slope −275.8 discharges/year; 95% CI −689.9 to 138.4; *p* = 0.1476).

### 2.3. Incidence of Staphylococcus aureus Stratified by Resistance Phenotype, Specimen Type, and Department

**Figure 4:**
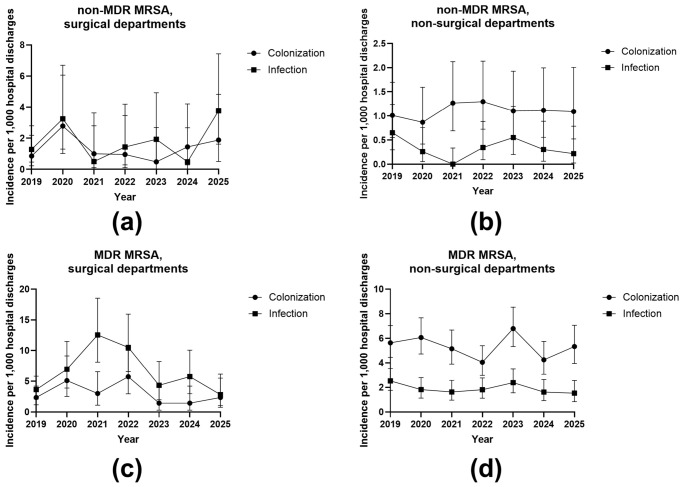
Incidence of methicillin-resistant *Staphylococcus aureus* (MRSA) per 1000 completed hospital discharges. (**a**) Non-MDR MRSA in surgical departments stratified by colonization and infection; (**b**) non-MDR MRSA in non-surgical departments; (**c**) MDR MRSA in surgical departments; (**d**) MDR MRSA in non-surgical departments between 2019 and 2025.

**Figure 5:**
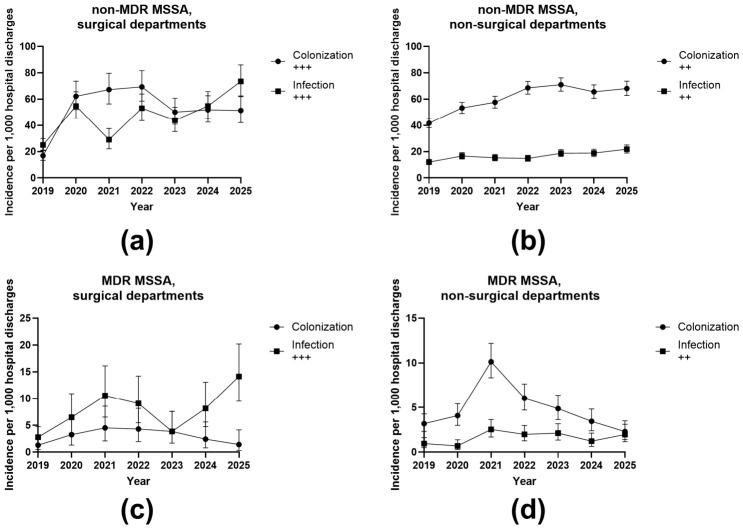
Incidence of methicillin-susceptible *Staphylococcus aureus* (MSSA) per 1000 completed hospital discharges. (**a**) Non-MDR MSSA in surgical departments stratified by colonization and infection; (**b**) non-MDR MSSA in non-surgical departments; (**c**) MDR MSSA in surgical departments; (**d**) MDR MSSA in non-surgical departments between 2019 and 2025. The symbols “++” and “+++” indicate the significance of change, where “++” denotes a moderate/significant change and “+++” denotes a highly significant change.

Figure 4 and Figure 5 show incidence trends of *S. aureus* per 1000 completed hospital discharges, stratified by methicillin resistance phenotype, multidrug resistance (MDR) status, clinical status, and department type. Overall, MSSA incidence was higher than MRSA incidence across all strata. Exact yearly values and trend estimates are provided in the Supplementary Materials—Figure S1: Supplementary trends table. The table shows detailed IRR, CI, *p*-values and significance for each, MRSA and MSSA trends.

#### 2.3.1. MRSA Incidence Trends

As shown in Figure 4, MRSA incidence remained low throughout the study period. For non-MDR MRSA in surgical departments (Figure 4a), colonization ranged from approximately 0.5 to 2.8 per 1000 discharges, and infection ranged from 0.4 to 3.8 per 1000. For non-MDR MRSA in non-surgical departments (Figure 4b), colonization ranged from approximately 0.9 to 1.3 per 1000, whereas infection ranged from 0.0 to 0.6 per 1000. For MDR MRSA in surgical departments (Figure 4c), colonization ranged from approximately 1.4 to 5.6 per 1000 discharges, and infection ranged from 2.4 to 12.5 per 1000. For MDR MRSA in non-surgical departments (Figure 4d), colonization ranged from approximately 4.0 to 6.8 per 1000, whereas infection ranged from 1.5 to 2.4 per 1000. According to the Poisson trend analysis summarized in Figure S1 none of the MRSA strata showed a statistically significant temporal trend. Annual incidence rate ratios ranged from 0.919 to 1.078, and all *p*-values were non-significant.

#### 2.3.2. MSSA Incidence Trends

As shown in Figure 5, MSSA incidence was substantially higher than MRSA incidence in all analyzed strata. For non-MDR MSSA in surgical departments (Figure 5a), colonization ranged from approximately 17 to 69 per 1000 discharges, and infection ranged from 25 to 73 per 1000. For non-MDR MSSA in non-surgical departments (Figure 5b), colonization ranged from approximately 42 to 71 per 1000, whereas infection ranged from 12 to 21 per 1000. For MDR MSSA in surgical departments (Figure 5c), colonization ranged from approximately 1.3 to 4.4 per 1000 discharges, and infection ranged from 3.7 to 14.2 per 1000. For MDR MSSA in non-surgical departments (Figure 5d), colonization ranged from approximately 2.1 to 10.1 per 1000, whereas infection ranged from 0.7 to 2.4 per 1000. Poisson trend analysis showed significant increases in several MSSA strata. In non-MDR MSSA, surgical departments, both colonization and infection increased significantly (IRR 1.104 and 1.147 per year, respectively; both *p* < 0.001). In non-MDR MSSA, non-surgical departments, both colonization and infection also increased significantly (IRR 1.077 and 1.083; both *p* < 0.001). In MDR MSSA surgical departments, infection increased significantly (IRR 1.192; *p* < 0.001), whereas colonization did not. In MDR MSSA non-surgical departments, infection increased significantly (IRR 1.097; *p* = 0.040), whereas colonization did not.

## 3. Discussion

This study suggests a sustained increase in the incidence of multidrug-resistant *Staphylococcus aureus* (MDR *S. aureus*), including methicillin-resistant *S. aureus* (MRSA), that emerged during the COVID-19 pandemic and continued into the post-pandemic period within this hospital-based dataset. This longitudinal analysis, based on routine laboratory data normalized to hospital activity, revealed distinct temporal patterns across resistance phenotypes, clinical presentation, and department types. Importantly, interpretation of these trends must consider substantial changes in both microbiological sampling and hospital activity during the study period. While many studies have focused on the early pandemic phase, our findings suggest that these effects may extend beyond the acute period, highlighting their potential longer-term clinical and organizational relevance [26,27,28].

The number of unique *S. aureus* isolates remained relatively stable between 2019 and 2021, followed by a pronounced increase in 2022 and a subsequent decline to a minimum in 2024, with partial recovery in 2025. A similar pattern was observed for antibiotic susceptibility testing (AST) measurements. In parallel, hospital activity decreased markedly after 2019 and remained persistently lower throughout 2020–2024. These shifts emphasize the importance of normalizing incidence to completed hospitalizations, as absolute isolate counts alone would not accurately reflect underlying epidemiological dynamics.

After normalization, clear differences emerged between MRSA and MSSA incidence. MSSA incidence was consistently higher across all strata, particularly for non-MDR strains and colonization, whereas MRSA incidence remained comparatively low but more variable over time. These findings are consistent with the known epidemiology of *S. aureus*, where methicillin-susceptible strains dominate overall burden, while resistant strains contribute disproportionately to severe healthcare-associated infections.

MRSA incidence demonstrated pronounced temporal variability, with increased MDR *S. aureus* incidence—particularly MRSA—during the pandemic period, followed by persistence into the post-pandemic phase. These trends may reflect the substantial strain placed on healthcare systems during COVID-19, including increased patient burden, prolonged hospital stays, and higher risk of healthcare-associated infections (HAIs). Similar increases in MRSA bacteremia and other HAIs during the pandemic have been reported in previous studies [26,27,28]. However, given the observational nature of this study, these associations should be interpreted cautiously.

In contrast, MSSA incidence exhibited more stable and structured patterns. In surgical departments, non-MDR MSSA colonization remained consistently high throughout the study period, while infection incidence showed greater temporal variability, including an increase toward the end of the observation period. In non-surgical departments, MSSA colonization increased steadily from 2019 to 2023 and remained elevated thereafter, whereas infection incidence remained lower but showed a gradual upward trend. Across all strata, colonization exceeded infection incidence, highlighting the substantial reservoir of asymptomatic carriage.

MDR MSSA incidence remained relatively low compared with non-MDR MSSA but demonstrated notable temporal fluctuations. In surgical departments, infection incidence increased again toward the end of the study period, while in non-surgical departments, colonization showed a transient peak followed by a gradual decline. These findings suggest dynamic changes in resistance patterns that may be influenced by local antimicrobial pressure and infection control practices.

The temporal overlap between increased MDR incidence and the COVID-19 period is consistent with reports linking pandemic-related healthcare disruption to changes in antimicrobial resistance patterns. Increased empirical antibiotic use, disruptions in infection prevention and control (IPC) practices, and reorganization of healthcare services may have contributed to these trends. However, published data remain heterogeneous, with some studies reporting increases in MRSA and others observing stable or reduced incidence depending on local conditions [29,30].

Notably, our findings indicate that elevated MDR *S. aureus* incidence persisted beyond the acute pandemic phase. This may reflect delayed recovery of antimicrobial stewardship programs, workforce shortages, and sustained pressure on healthcare systems [31]. The reorganization of care, including the establishment of COVID-19 units, patient cohorting, and staff redeployment, likely further disrupted standard IPC practices. Although enhanced use of personal protective equipment (PPE) was effective for respiratory pathogens, it may not have fully addressed transmission of contact-mediated organisms such as MRSA [32].

Antibiotic prescribing practices during the pandemic also played a crucial role in shaping the dynamics of antimicrobial resistance (AMR). National and international guidelines consistently recommend that antibiotics should be reserved for patients with clear evidence of bacterial co-infection, with the aim of avoiding prophylactic use in COVID-19 cases [33]. Our findings align with this, showing that such antibiotic overuse likely exerted selective pressure on *S. aureus* populations, promoting the persistence of multidrug-resistant strains. The widespread use of antibiotics during this period may have contributed to the sustained elevated incidence of MRSA, which is expected to remain a significant issue for healthcare settings long after the resolution of the pandemic, [34,35], favoring the persistence of multidrug-resistant *S. aureus* (MDR *S. aureus*) and methicillin-resistant *S. aureus* (MRSA) in the post-pandemic period [36]. Notably, hospital-onset MRSA bloodstream infections remained elevated beyond the first year of the pandemic [36,37].

As with previous studies, our analysis shows that MRSA infections are associated with elevated healthcare costs compared to infections caused by methicillin-susceptible *S. aureus* (MSSA) [38,39]. Additionally, at the institutional level, sustained increases in MRSA burden can strain patient flow, necessitate enhanced isolation measures, and consume limited hospital resources. These issues are especially impactful during periods of healthcare system recovery, such as post-crisis or pandemic situations [39].

According to the European Centre for Disease Prevention and Control (ECDC) Surveillance Atlas, MRSA incidence and proportions have generally declined or stabilised across the EU in recent years. However, substantial inter-country variability persists, and Slovakia demonstrates fluctuating MRSA trends without a consistent long-term decrease. These observations suggest that, while our findings reflect local hospital dynamics, they may also be consistent with the relatively high and variable antimicrobial resistance burden reported in Slovakia. As ECDC surveillance primarily focuses on MRSA, MSSA trends are not reported separately and represent the majority of *S. aureus* isolates [40].

Overall, these findings highlight the complex interplay between healthcare system factors, antimicrobial use, and pathogen dynamics. The consistently higher incidence observed in surgical departments underscores the importance of targeted surveillance and infection control strategies in high-risk clinical settings. The predominance of colonization over infection further emphasizes the need to consider asymptomatic carriage as a key component of *S. aureus* epidemiology.

Despite these insights, several limitations should be acknowledged. This study was based on retrospective microbiological laboratory data. Due to the irreversible anonymization of the dataset, it was not possible to perform a manual review of individual medical records to verify the clinical status of each patient. Consequently, the reported rates of infection and carriage represent a structured approximation rather than a clinically confirmed diagnosis. Changes in EUCAST breakpoints over time may have introduced variability, and department classification based on internal coding (P-codes) may be subject to minor misclassification.

Despite this, the use of standardized, routinely collected laboratory data represents a robust and scalable approach for longitudinal surveillance. By integrating microbiological data with hospital activity metrics, this study provides a realistic representation of antimicrobial resistance dynamics in a hospital setting. These findings support the need for continuous, hospital-level surveillance and adaptive infection control strategies to mitigate the long-term impact of antimicrobial resistance of *S. aureus* and its resistance trends in a real-world hospital setting, which is essential for guiding local antimicrobial stewardship programs.

## 4. Materials and Methods

### 4.1. Data Source

The primary data source comprised records from the laboratory information system (LIS). These unstructured diagnostic data included microorganism identification by the MALDI TOF-MS method, and antimicrobial susceptibility testing was performed using routine diagnostic systems available in participating microbiology laboratories. Interpretation of susceptibility results was performed according to the European Committee on Antimicrobial Susceptibility Testing (EUCAST) guidelines. EUCAST breakpoint criteria were updated and implemented annually in accordance with official EUCAST revisions during the study period. An expert microbiological system enabling ‘interpreted reading’ was used to support the detection of common resistance mechanisms and ensure consistency of interpretation. Antimicrobial susceptibility testing was performed using routine diagnostic panels adapted to the microorganism and specimen type. These panels included a broad range of clinically relevant antibiotics. For example, Gram-positive isolates were routinely tested against antibiotics such as oxacillin/cefoxitin, vancomycin, clindamycin, erythromycin, and linezolid, while Gram-negative isolates were tested against beta-lactams (e.g., cefotaxime, ceftazidime, piperacillin/tazobactam), carbapenems (e.g., meropenem), aminoglycosides (e.g., gentamicin, amikacin), and fluoroquinolones (e.g., ciprofloxacin).

Microbiological data were obtained from the laboratory information system, used in accredited microbiology laboratories in Slovakia. These laboratories participate in external quality assessment programmes (e.g., INSTAND e.V. and national reference center schemes). As this is an institutional database, it is not publicly available.

### 4.2. Database

The data used in this study originated from the expert medical information system AMEBA. The Ameba laboratory information system, implemented in routine microbiological practice since approximately 2018 based on institutional records, was used for data management and analysis. AMEBA (version 3.0) is an advanced software platform designed for real-time integration, interpretation, and visualization of microbiological laboratory results.

The system collects unstructured diagnostic data from microbiological laboratories (including MALDI-TOF MS identification and antibiotic susceptibility tests), which are then processed using machine learning algorithms to extract relevant information. All data were collected and maintained in Slovak and English to enable consistency in international reporting and future interoperability.

### 4.3. Data Extraction

The defined years for the analysis were from 2019 to 2025. Microbiological data were extracted from Ameba. The system allows dynamic updating of results for a given isolate as additional testing or interpretative changes are performed. For the purposes of this study, only the final result validated by a clinical microbiologist was included in the analysis.

To avoid duplication, records were filtered using a unique protocol identifier, ensuring that each isolate was represented only once and that interim or repeated entries were excluded. Each microbiological sample was linked to the requesting hospital department using internal departmental identification codes (‘P codes’) derived from the hospital information system assigned by the Health Care Surveillance Authority (Úrad pre dohľad nad zdravotnou starostlivosťou, Slovakia). The P-code typically consists of a fixed-length numeric string. The full P-code enables hierarchical identification at three levels: provider entity (the initial 8 digits), medical specialization (commonly a 3–4-digit code), and individual workplace (typically a 2-digit suffix).

Multidrug resistance was defined according to Magiorakos et al. (2012) [41]. Outpatient departments were excluded, and only inpatient units were analysed, which improves population homogeneity but may limit the generalizability of the findings to outpatient settings. For analytical purposes, departments were further grouped into surgical and non-surgical categories based on their clinical profile.

### 4.4. Data Cleaning and Preprocessing

Microbiological datasets were processed using the Konstanz Information Miner (KNIME) Analytics Platform (v5.4.3). Data integration pipelines were developed to harmonize sample metadata, antimicrobial susceptibility profiles, and hospital department identifiers across a five-year retrospective window. Preprocessing included dataset merging, standardization of susceptibility interpretations according to EUCAST criteria, classification of ward types via P codes, and consistency checks for duplicate or incomplete records. All KNIME workflows used in this phase are provided as Figure S1 to ensure full reproducibility.

The analytical pipeline performs automated data cleaning, deduplication, and harmonization of antimicrobial susceptibility results in accordance with EUCAST criteria. Isolates were subsequently classified by resistance phenotype, with particular focus on multidrug-resistant (MDR) *S. aureus* and methicillin-resistant *S. aureus* (MRSA). The assignment of isolates to colonization or infection categories was performed automatically within the KNIME workflow based on predefined classification rules. These rules were developed and validated by infectious disease specialists and hospital epidemiologists, who defined the criteria for assigning individual specimen types and clinical contexts to colonization or infection. Once implemented, the workflow applied these expert-derived rules consistently across the entire dataset, ensuring reproducible and unbiased classification.

Similarly, clinical departments were clustered into surgical and non-surgical categories using expert-defined institutional criteria. These categorization rules, reflecting differences in patient populations, care pathways, and exposure to invasive procedures, were encoded directly into the KNIME workflow. This approach enabled automated, standardized department-level stratification while preserving clinical relevance.

A central feature of the workflow is automated temporal aggregation and normalization of resistance incidence metrics to completed hospitalizations, ensuring robust comparability across calendar years and department types despite substantial changes in hospital activity and capacity over time. All steps of data translation, standardization, filtering, and aggregation were implemented using KNIME Base nodes complemented by selected extensions, including KNIME AI Extension, KNIME Excel Support, KNIME Expressions, KNIME Javasnippet, KNIME JSON-Processing, KNIME Math Expression (JEP), KNIME Python Integration, KNIME Quick Forms, KNIME Views, and Vernalis KNIME Nodes. Together, these components form a fully reproducible analytical pipeline that underpins all downstream results presented in this study.

Classification of isolates as originating from surgical versus non-surgical wards, as well as the interpretation of results in the context of infection versus colonization, were based on definitions and operational criteria established by a multidisciplinary team of infectious disease specialists and hospital epidemiologists. As the dataset was irreversibly anonymized at the source to comply with GDPR and institutional ethical standards, a manual retrospective review of individual patient medical records was not feasible. To address this, robust classification logic was adopted based on the anatomical site of the specimen, following the principles of the European Antimicrobial Resistance Surveillance Network (EARS-Net) [42]. In accordance with EARS-Net protocols, all isolates from primarily sterile sites, such as blood and cerebrospinal fluid (CSF), were classified as invasive infections [2,43]. We further extended this logic to include other clinically significant specimens obtained via invasive procedures, such as bronchoalveolar lavage (BAL) and deep abscess drainage, which were categorized as infections due to their high predictive value for active disease [44,45]. Conversely, isolates from known carriage sites, including nasopharyngeal and oropharyngeal swabs, were classified as colonization, reflecting the high prevalence of asymptomatic *S. aureus* carriage in the hospital population [41,46]. These definitions reflect routine clinical and epidemiological practice within the institution. Given the retrospective design, the study deliberately leveraged available real-world data to evaluate their suitability and validity for longitudinal surveillance and epidemiological analyses.

### 4.5. Data Analysis and Visualization

The processed dataset was analyzed using KNIME through a series of visual and statistical workflow components. The core analyses focused on comparing counts of multiresistant versus susceptible *Staphylococcus* isolates over time, stratified by predefined time periods. Aggregation and grouping operations were applied to compute frequency distributions, followed by visualization using categorical bar plots to illustrate temporal trends. Visual outputs were generated directly within KNIME using bar chart nodes configured with “TimePeriod” as the category axis and multiple frequency columns (e.g., “Multiresistant + Count (Pathogen)”, “Susceptible + Count (Pathogen)”). These plots supported rapid interpretation of resistance evolution across clinical departments. Once the input data were standardized, the downstream analytical part of the workflow was applied uniformly. This second part, consisting of Incidence Graph Prep, Poisson log-link model, and Figure S1, served as a reusable analytical module. In this section, annual counts were aggregated, incidence rates were normalized to completed hospitalizations, and temporal trends were evaluated using a Poisson log-link model. Figure S1 containing exact yearly values were generated in parallel.

Outputs generated in KNIME were subsequently exported to GraphPad Prism (version 11.0.1.), where final publication-ready versions of the figures were prepared.

Significant temporal trends are presented directly in the main text and figures, including incidence rate ratios (IRRs) with corresponding 95% confidence intervals. Complete results, including both significant and non-significant trends, are provided in Figure S1.

All workflow logic, including node configurations and output mappings, is available as Figure S1 to ensure full reproducibility and transparency. See whole process: https://hub.knime.com/medirex%20group%20academy%20n.o.%20team/spaces/Public/~WsAMttojPfCUaZQW/ (accessed on 10 January 2026).

## 5. Conclusions

In summary, this study demonstrates that the COVID-19 pandemic was associated with a sustained increase in MDR methicillin-susceptible *S. aureus* and MRSA incidence that persisted into the post-pandemic period. These findings highlight that short-term crisis responses may have lasting consequences for antimicrobial resistance control when core infection prevention and control (IPC) and antimicrobial stewardship activities are disrupted. This retrospective analysis establishes a data-driven baseline for *S. aureus* surveillance. The subsequent integration of whole-genome sequencing represents the second step toward comprehensive molecular epidemiology, supporting improved infection control and antimicrobial resistance monitoring.

Future pandemic preparedness and emergency response strategies should therefore explicitly include safeguards to preserve these essential programs, ensuring that immediate crisis management does not result in prolonged setbacks in the control of MDR *S. aureus* and MRSA and, ultimately, compromise patient safety.

## Figures and Tables

**Table 1:** Annual hospital AST measurements.

Year	Unique *S. aureus* Isolates, *n*	AST Measurements, *n*	Non-Surgical Discharges, *n*	Surgical Discharges, *n*	Total Discharges, *n*
2019	1152	17,087	13,839	4675	18,514
2020	1177	17,063	11,530	2154	13,684
2021	1180	17,841	11,061	1991	13,052
2022	1384	19,487	11,589	2089	13,678
2023	1316	17,982	10,886	2076	12,962
2024	1120	14,460	9865	2082	11,947
2025	1151	13,062	9174	2121	11,295

## Data Availability

Restrictions apply to the availability of these data. Data were obtained from Neoxx JSC and are available with the permission of Neoxx JSC.

## Supplementary Materials

The following supporting information can be downloaded at: https://www.mdpi.com/article/10.3390/antibiotics15050443/s1, Figure S1: Supplementary trends table.

## Abbreviations

The following abbreviations are used in this manuscript:

AMR	Antimicrobial resistance
HIC	High-income countries
LMIC	Low- and middle-income countries
LIS	Laboratory information system
MSSA	Methicillin-sensitive *S. aureus*
MRSA	Methicillin-resistant *S. aureus*
MDR	Multidrug resistant
ECDC	European Centre for Disease Prevention and Control
KNIME	Konstanz Information Miner
EUCAST	European Committee on Antimicrobial Susceptibility Testing
PPE	Personal protective equipment
