# Supplementary material for: Monitoring the Burden of Staphylococcus aureus: A Multi-Year Retrospective Study Using Routine Laboratory Data from a Slovak Hospital
Source: Antibiotics (Basel). 2026 Apr 29;15(5):443. doi: 10.3390/antibiotics15050443 (PMC13203488; doi:10.3390/antibiotics15050443)
Supplement: Supplementary file 1 [file antibiotics-15-00443-s001.zip › antibiotics-4187588-supplementary.pdf]

Trend symbol legend: ns = not significant; + / - = small change (<5% per year); ++ / -- = moderate change (5–10% per year); +++ / --- = large change (>10% per year), assigned only when p < 0.05.

Group	Clinical status	IRR per year	95% CI	p-value	Significant trend	Direction	Trend symbol	Trend class
**non-MDR MRSA, surgical departments**	Colonization	1.029	0.847–1.249	0.774	No	No significant trend	**ns**	No significant trend
4a	Infection	1.078	0.914–1.273	0.373	No	No significant trend	**ns**	No significant trend
**non-MDR MRSA, non-surgical departments**	Colonization	1.022	0.919–1.135	0.693	No	No significant trend	**ns**	No significant trend
4b	Infection	0.919	0.758–1.114	0.390	No	No significant trend	**ns**	No significant trend
**MDR MRSA, surgical departments**	Colonization	0.940	0.825–1.072	0.357	No	No significant trend	**ns**	No significant trend
4c	Infection	0.985	0.901–1.077	0.738	No	No significant trend	**ns**	No significant trend
**MDR MRSA, non-surgical departments**	Colonization	0.981	0.935–1.029	0.427	No	No significant trend	**ns**	No significant trend
4d	Infection	0.949	0.876–1.029	0.205	No	No significant trend	**ns**	No significant trend
**non-MDR MSSA, surgical departments**	Colonization	1.104	1.069–1.140	<0.001	Yes	Increasing	**+++**	Large increase
5a	Infection	1.147	1.109–1.185	<0.001	Yes	Increasing	**+++**	Large increase
**non-MDR MSSA, non-surgical departments**	Colonization	1.077	1.062–1.093	<0.001	Yes	Increasing	**++**	Moderate increase
5b	Infection	1.083	1.054–1.113	<0.001	Yes	Increasing	**++**	Moderate increase
**MDR MSSA, surgical departments**	Colonization	1.033	0.905–1.180	0.628	No	No significant trend	**ns**	No significant trend
5c	Infection	1.192	1.096–1.295	<0.001	Yes	Increasing	**+++**	Large increase
**MDR MSSA, non-surgical departments**	Colonization	0.956	0.908–1.005	0.079	No	No significant trend	**ns**	No significant trend
5d	Infection	1.097	1.004–1.197	0.040	Yes	Increasing	**++**	Moderate increase

Figure S1: Supplementary trends table
